# LncRNA XIST promotes myocardial infarction by regulating *FOS* through targeting miR-101a-3p

**DOI:** 10.18632/aging.103072

**Published:** 2020-04-21

**Authors:** Bin Lin, Jing Xu, Feng Wang, Jiaxiang Wang, Hui Zhao, Deguang Feng

**Affiliations:** 1 Department of Cardiovascular Surgery, The First Affiliated Hospital of Zhengzhou University, Zhengzhou 450052, Henan, China

**Keywords:** lncRNA XIST, miR-101a-3p, FOS, NMCMs, myocardial infarction

## Abstract

The purpose of this study was to reveal the hypothesis that lncRNA X inactive specific transcript (XIST) can participate in the regulation of cardiomyocyte apoptosis in neonatal mice cardiomyocytes (NMCMs) and myocardial infarction (MI) through targeting miR-101a-3p. NMCMs were isolated from neonatal C57BL/6 mice and anoxia was induced in hypoxic chamber. MTT assay and flow cytometry were used to determine proliferation and apoptosis respectively. The target relationship among XIST, miR-101a-3p and *FOS* was revealed by bioinformatic analysis, luciferase reporter assay, pull-down assay and RNA immunoprecipitation assay. The expression of XIST, miR-101a-3p, *FOS* and apoptosis-related proteins was determined by qRT-PCR or western blot. MI model was constructed to reveal the role of XIST. We found that XIST was up-regulated in NMCMs under anoxia condition. Moreover, XIST increased FOS expression by sponging miR-101a-3p in anoxia cells. Silencing XIST expression improved cell viability and suppressed apoptosis in vitro and inhibited myocardial infarction by reducing the level of c-FOS and apoptosis-related proteins *in vivo*. Our findings suggest that XIST is involved in MI, modulation of its level can be used as a new strategy or potential target in the treatment of myocardial infarction.

## INTRODUCTION

Coronary heart disease (CHD), also called coronary artery disease (CAD) [1], is the shortened name of coronary atherosclerotic heart disease. CHD is a common chronic disease, which is mainly is mainly due to the gradual constriction of the blood vessels that supply oxygenated blood to the myocardium [2]. Coronary heart disease includes a group of diseases, including myocardial infarction (MI), unstable angina, stable angina and sudden cardiac death [3]. MI is the material cause of mortality in CHD patients, it is the leading global cause of death, especially in China, and its incidence and fatality rate have increased [4]. Epidemiological studies of MI demonstrated that age, male gender, smoking, elevated blood pressure, diabetes, obesity and a sedentary lifestyle led to higher risk of MI occurrence [5]. Thus, effective prevention and treatment of MI is an important topic. Recently, with the progress of genetic sequencing, an increasing number of disease-related genes have been found, which will help people understand the relationship between genes and diseases [6, 7].

Long non-coding RNAs (LncRNAs), defined as RNAs that is more than 200 nucleotides in length, a variety of lncRNAs could play dynamic roles in transcriptional and translational regulation [8]. Recent studies have shown that lncRNAs play a crucial role in cardiovascular diseases [9, 10]. For example, Wang et al. has authenticated the cardiac apoptosis-related lncRNA (CARL) as a molecular sponge for miR-539, which promoted mitochondrial fission and apoptosis by disrupting the function of prohibitin-2 (PHB2) [11]. Moreover, a cardiac fibroblast-enriched lncRNA denominated WISPER (WIsp2 Super-Enhancer-associated RNA) was found to promote MI-induced cardiac fibrosis [12]. More interestingly, regulatory networks between miRNAs and lncRNAs have recently been discovered, these results provide insights into the function of non-coding RNA and its potential as a treatment for disease [13]. The lncRNA XIST (X-inactive specific transcript) is produced by XIST gene and the master regulator of X inactivation in mammals [14]. More and more studies have shown that lncRNA XIST plays critical role in cell proliferation, differentiation, and genome maintenance [15, 16]. However, it remains unclear whether lncRNAs especially XIST participate in the regulation of myocardial infarction, the molecular mechanism and pathway of myocardial infarction need further study, which may have clinical significance.

MicroRNAs (miRNAs) are evolutionarily conserved, small single-stranded non-coding RNAs (approximately 22 nucleotides), which play pivotal roles in regulating gene expression [17]. Through binding to the 3’-untranslated region (3’-UTR) of mRNAs, miRNAs are able to affect the stability and translation of mRNAs [18]. More and more evidence shows that miRNAs participate in the regulation of development, differentiation, proliferation and apoptosis and cell conversion [19]. Several miRNAs are related to MI. MiR-21 effectively restores cardiac function after myocardial infarction [20]. Knocking down E2F1 suppresses mitochondrial fragmentation, apoptosis and myocardial infarction by affecting miR-421 levels [21]. Yang et al. demonstrated that miRNA-101 inhibited postinfarct cardiac fibrosis and improved Left Ventricular Compliance via the FOS/TGFβ1 pathway [22]. However, how MI is regulated by miRNAs especially miR-101a-3p is still not yet clear.

Our present work reveals that FOS is a target of miR-101a-3p and miR-101a-3p participates in the suppression of FOS translation. MiR-101a-3p reduce myocardial infarction and protects myocardial cell apoptosis through targeting FOS *in vivo* and *in vitro*. Furthermore, we identified that the lncRNA X inactive specific transcript (XIST), directly binds to miR-101a-3p and therefore inhibits its activity. XIST regulates FOS expression and the consequent myocardial infarction through miR-101a-3p. To put it in a nutshell, XIST regulates myocardial infarction and myocardial cell apoptosis through targeting the miR-101a-3p/FOS pathway. Our data provide new clues on the understanding of the regulatory mechanism between lncRNAs and miRNAs in molecular regulation of myocardial infarction and myocardial cell apoptosis.

## RESULTS

### Upregulation of XIST in infarcted heart and neonatal mice cardiomyocytes model of anoxia

**Figure 1:**
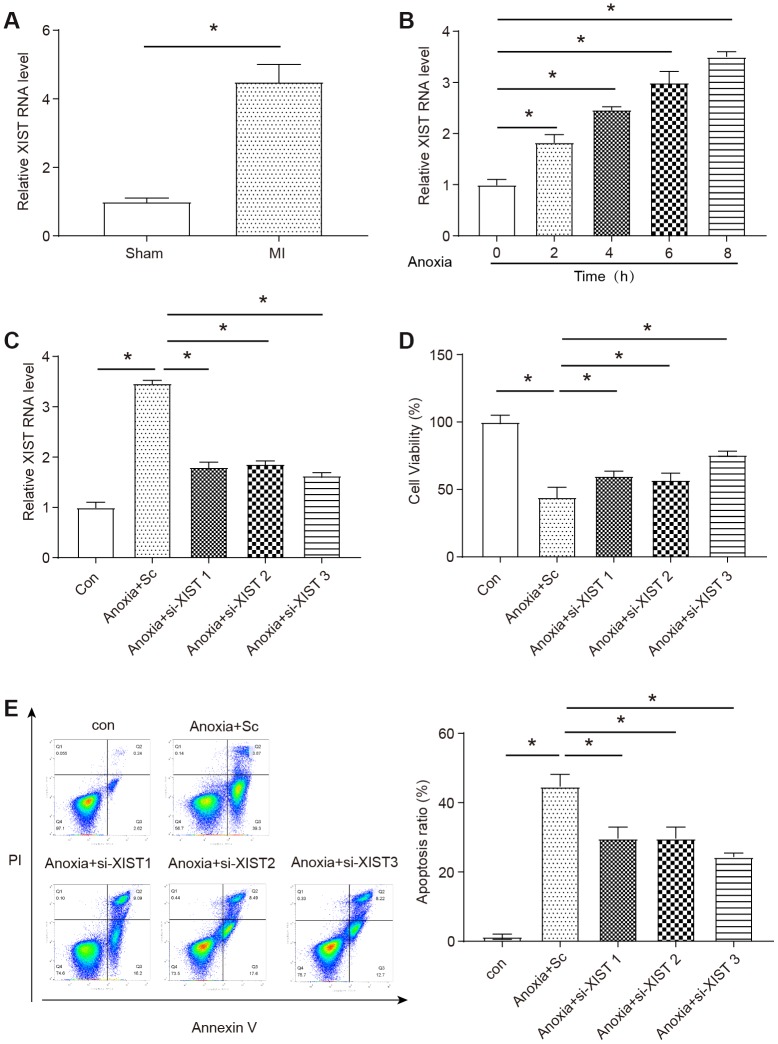
**Expression levels of a long non-coding RNA XIST and the role in neonatal mice cardiomyocytes model of anoxia.** (**A**) The level of XIST was measured by qRT-PCR in the infarct zone of mice MI hearts compared to a sham group (n=6). The expression of XIST was significantly upregulated in MI hearts. (**B**) The level of XIST was measured by qRT-PCR in NMCMs treated with anoxia for 0-8 h (n=3). The expression of XIST was significantly upregulated in NMCMs under anoxia condition. (**C**) The level of XIST RNA in NMCMs after transfected with XIST siRNAs (S1-S3) and siRNA control. All XIST siRNAs successfully knocked down the expression of XIST (n=3). (**D**) Cell viability was detected using the MTT assay (n=3). (**E**) Apoptotic cells were analyzed by flow cytometry after anoxia treatment (n=3). Anoxia condition was induced by placing cells in hypoxia chamber for 8 h. Control: normal culture, Sc: the siRNA control. Data are shown as mean ± SD, * *P<0.05.*

Using a murine model of myocardial infarction, we first evaluated the level of the XIST by qRT-PCR in mice heart samples. After 24 h of myocardial infarction, the level of XIST was significantly increased in the infarct area (P<0.05, Figure 1A). Neonatal mouse cardiomyocytes (NMCMs) were isolated from neonatal mice and the XIST expression level in NMCMs kept increasing with time under anoxia condition (P<0.05, Figure 1B).

### Knocking down XIST protects cardiomyocyte viability and prevents apoptosis

To identify the role of XIST played under anoxia condition, specific XIST-siRNAs (S1-S3) were applied to interfere with the expression of XIST. As shown in Figure 1C, S1-S3 all successfully silenced the expression of XIST compared to siRNA control (Sc) under the condition of anoxia (P<0.05), the XIST-siRNA 3 was used in further studies. We used flow cytometry and MTT assay to determine the effect of XIST on NMCMs under anoxia condition. Obviously, knocking down of XIST significantly improved the cell viability and decreased apoptosis rate under anoxia condition (P< 0.05, Figure 1D,1E).

### XIST is able to directly bind to miR-101a-3p

**Figure 2:**
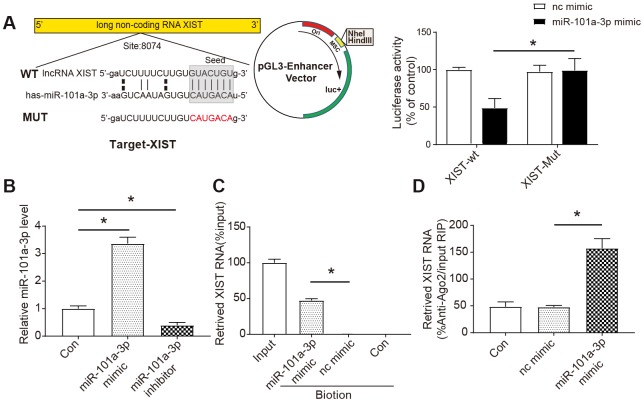
**The interaction between XIST and miR-101a-3p.** (**A**) Bioinformatic analysis (StarBase and miRbase) indicated that miR-101a-3p was a target of XIST (left panel) and the luciferase activity was analysed (right panel) (n=3). XIST-WT: wild-type XIST, XIST-Mut: XIST with mutated 3’-UTR. (**B**) In vitro transfection efficiency was determined by measuring miR-101a-3p and miR-101a-3p inhibitor using qRT-PCR (n=3). (**C**) The results of RNA pull-down assay. NMCMs cell lysates were incubated with biotin-labeled miR-101a-3p, after pull-down, microRNAs was extracted and analysed by qRT-PCR (n=3). (**D**) The results of RNA immunoprecipitation assay. Anti-AGO2 RIP was performed in NMCMs cells transiently overexpressing miR-101a-3p mimic or nc mimic, followed by qRT-PCR to detect XIST associated with AGO2 (n=3). nc mimic: a sequence did not interact with XIST; con: control. Data are shown as mean ± SD, * *P<0.05.*

We employed the luciferase assay system to test whether XIST could influence the expression of miR-101a-3p. Based on bioinformatics databases (StarBase and miRbase), we identified a potential target relationship between 3’-UTR of lncRNA XIST and miR-101a-3p. The luciferase reporter assay revealed that the wild-type 3’-UTR of XIST exhibited a low translation level in the presence of miR-101a-3p, whereas the mutated 3’-UTR did not show a significant response to miR-101a-3p (Figure 2A). NMCMs were transfected with miR-101a-3p mimic or miR-101a-3p inhibitor to verify the efficiency of overexpression or knock down (Figure 2B). Furthermore, RNA pull down assay was performed to extract RNA that interacts with miR-101a-3p and the level of XIST was assessed by qRT-PCR. The specific binding between miR-101a-3p and XIST was further verified by affinity pull-down of XIST through pull down assay. In the pull-down mixture, 48 % of XIST was detected in the miR-101a-3p mimic group, which was significantly higher than that in the nc mimic group (*P*<0.05, Figure 2C). The miRNAs are confirmed to bind their targets and lead to RNA degradation and/or translational inhibition in an AGO2-dependent manner [23]. To explore whether miR-101a-3p was modulated by XIST in such a way, anti-AGO2 RIP was performed in NMCMs transiently overexpressing miR-101a-3p, followed by qRT-PCR to detect XIST associated with AGO2. As shown in Figure 2E, the Ago2 RIP assay showed that XIST level in the miR-101a-3p group was significantly higher than that in the nc mimic group (*P*<0.05, Figure 2D). Based on these results, we concluded that XIST directly targets and binds to the miR-101a-3p.

### MiR-101a-3p participates in the regulation of FOS expression

**Figure 3:**
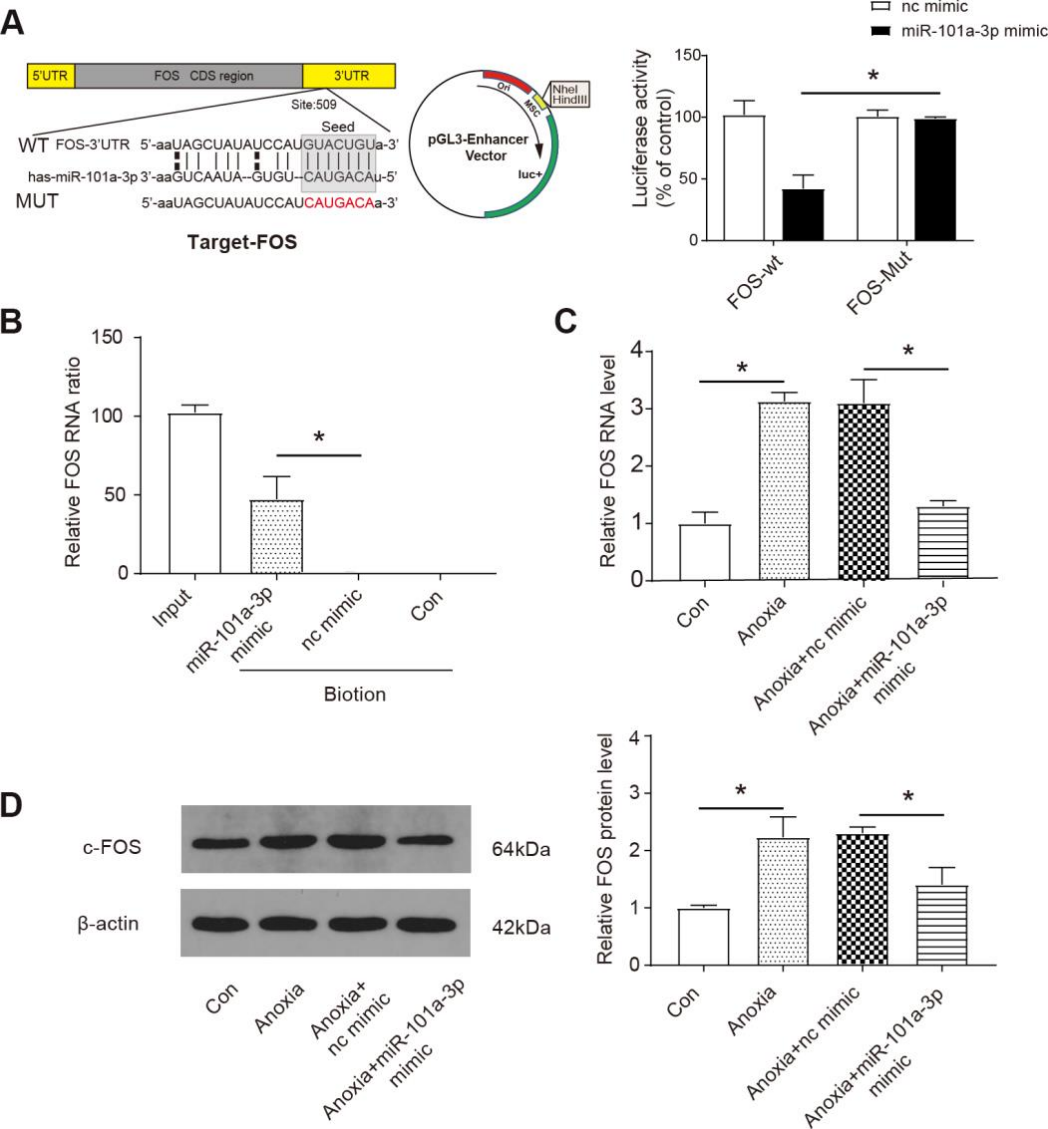
**MiR-101a-3p participates in the regulation of Fos expression.** (**A**) Bioinformatic analysis (http://www.microrna.org/) showed that *FOS* was a target of miR-101a-3p (upper panel) and the luciferase activity was analysed (lower panel) (n=3). *FOS*-WT: wild-type *FOS*, *FOS*-Mut: *FOS* with mutated 3’-UTR. (**B**) The results of RNA pull-down assay (n=3). Nc mimic: a sequence did not interact with *FOS*; Con: control. (**C**) The expression of *FOS* was measured by qRT-PCR in NMCMs under normal or anoxia condition. The application of miR-101a-3p mimic down-regulated *FOS* expression (n=3). (**D**) The protein level of c-Fos was measured by western blot in in NMCMs under normal or anoxia condition. The application of miR-101a-3p mimic down-regulated C-FOS expression (n=3). Data are shown as mean ± SD, * *P<0.05.*

Next, we aimed to identify the downstream target of miR-101a-3p. According to the bioinformatics databases (http://www.microrna.org/), we identified a potential target relationship between miR-101a-3p and *FOS*. The luciferase reporter assay revealed that the wild-type 3’-UTR of FOS exhibited a low translation level in the presence of miR-101a-3p, whereas the mutated 3’-UTR did not show a significant response to miR-101-3p (*P*< 0.05, Figure 3A). Moreover, the specific binding between miR-101a-3p and FOS was further verified by affinity pull-down of FOS through pull down assay. As our data shows, 51 % of *FOS* was detected in the pull-down mixture, which was significantly higher than that in nc mimic group (*P*< 0.05, Figure 3B). Confirming that *FOS* was a target of miR-101a-3p, we then explored the regulatory effect of miR-101a-3p on *FOS* by qRT-PCR. As shown in Figure 3C, the *FOS* RNA level was significantly upregulated under anoxia condition and application of miR-101a-3p mimic reversed the upregulation (*P*<0.05). The protein product of *FOS*, c-FOS, was measured by western blot. Similarly, the c-FOS level increased under anoxia condition and the upregulation was suppressed by miR-101a-3p mimic (*P*<0.05, Figure 3D). According to the above experimental data, we determined that miR-101a-3p participates in the regulation of FOS expression by targeting to miR-101a-3p.

### XIST regulates the expression of miR-101a-3p and FOS in NMCMs under anoxia condition

**Figure 4:**
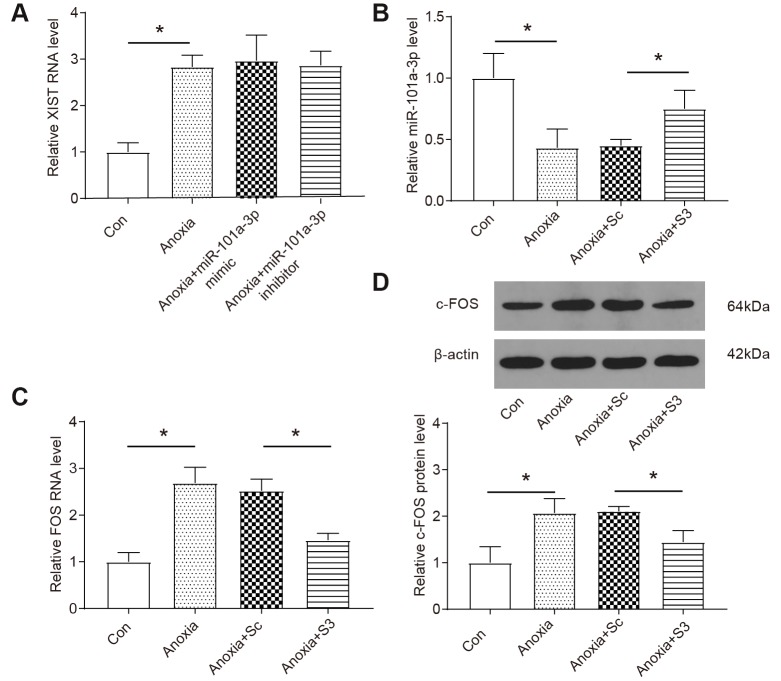
**XIST regulates the expressions of miR-101-3p and FOS in NMCM under anoxia condition.** (**A**) The expression of XIST was measured by qRT-PCR (n=3). NMCMs were transfected with miR-101a-3p inhibitor or mimic. (**B**) The expression of miR-101a-3p was measured by qRT-PCR. Knockdown of XIST increases the expression levels of miR-101a-3p under anoxia. NMCMS were infected with XIST-siRNA or XIST-sc (n=3). (**C**, **D**) The expression of *FOS* and c-FOS was measured by qRT-PCR and western blot. Knockdown of XIST decreases the expression levels of *FOS* and c-FOS under anoxia. NMCMS were infected with XIST-siRNA or XIST-sc (n=3). Cells were transfected with miR-101a-3p mimic, inhibitor, S3 or si-RNA control (Sc) and placed in hypoxia chamber for 8 h to induce anoxia condition. Data are shown as mean ± SD, * *P<0.05.*

Confirming the target relationship among XIST, miR-101a-3p and *FOS*, we then investigated how XIST regulated the expression of its downstream targets. As Figure 4A shows that the expression level of lncRNA XIST was significantly upregulated in NMCMs under anoxia condition and the up-regulation was not affected by application of miR-101a-3p mimic or inhibitor. In contrast, the level of miR-101a-3p was significantly reduced under anoxia condition and transfecting cells with XIST siRNAs (S3) significantly improved miR-101a-3p expression under anoxia condition (*P*<0.05, Figure 4B). On the other hand, the level of *FOS* mRNA was increased in anoxia cells and the upregulation was suppressed in the XIST siRNAs group under anoxia condition both at the protein and mRNA levels (*P*<0.05, Figure 4C, 4D). To sum up, the upregulation of XIST expression under anoxia condition promoted the *FOS* expression by targeting and suppressing the expression of miR-101a-3p.

### XIST regulates myocardial infarction and apoptosis through miR-101a-3p and *FOS*

**Figure 5:**
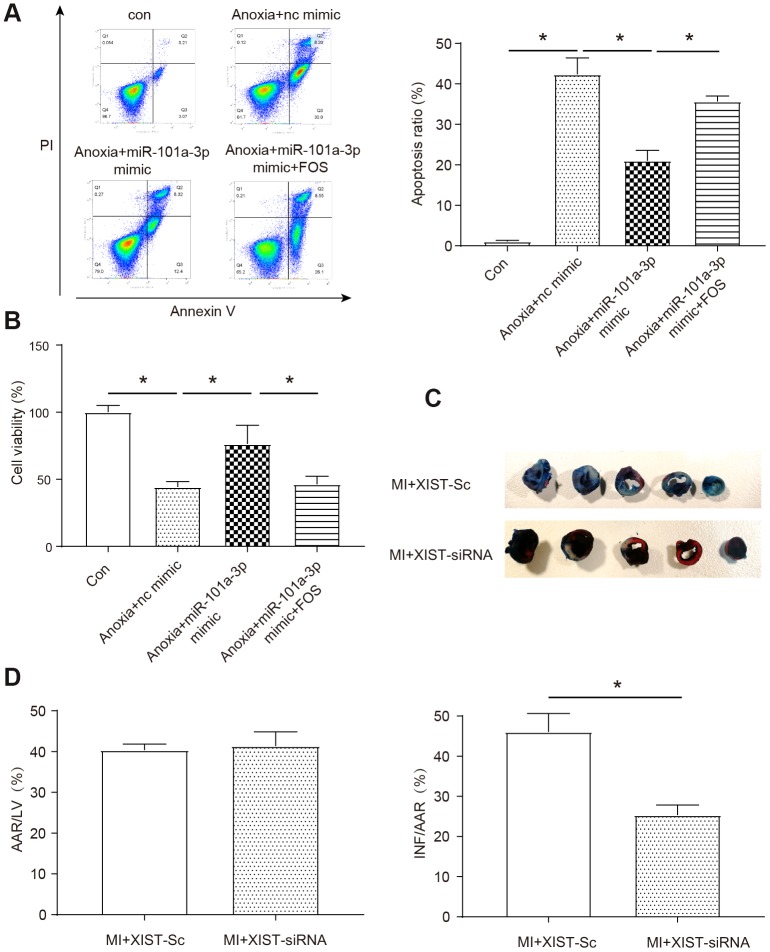
**XIST regulates myocardial infarction and apoptosis through miR-101a-3p and FOS.** (**A**) Apoptotic cells were analyzed by flow cytometry after anoxia treatment (n=3). (**B**) Cell viability was detected using the MTT assay. (**C**) The myocardial infarction size was measured upon MI of mice as determined by 2, 3, 5-triphenyltetrazolium chloride (TTC) staining (n=6). (**D**) The area at risk/left ventricle weight (AAR/LV) ratio and the infarct size/area at risk (INF/AAR) ratio were determined to evaluate the homogeneity of surgery and the severity of MI, respectively (n=6). Con: control, Adv-sc: Adv-control. Data are shown as mean ± SD, * *P<0.05*.

In order to explore whether the target genes miR-101a-3p and FOS in the downstream of XIST could affect anoxia-induced NMCMs apoptosis and cell viability, we transfected miR-101a-3p into NMCMs and overexpressed the FOS at the same time to determine whether miR-101a could directly regulate FOS to affect the apoptosis and viability of NMCMs. As shown in Figure 5A, the apoptosis rate was significantly increased under anoxia condition and application of miR-101a-3p mimic could protect cells against apoptosis in certain degree (P<0.05). Moreover, as assumed before, the application of FOS contradicted the protection effect of miR-101a-3p mimic (P<0.05). Similarly, anoxia significantly decreased the cell viability and miR-101a-3p mimic also had a protective effect (P<0.05, Figure 5B), at the same time *FOS* reversed the positive effect of miR-101a-3p to some extent. To understand the role of XIST in the mice model of myocardial infarction, we performed MI in the mice model and evaluated the effects of inhibiting XIST on cardiac damage by TTC staining (Figure 5C). Our results showed that the mice treated with XIST-siRNA adenoviruses exhibited a significant reduction in myocardial infarction sizes (INF/AAR) in response to MI as determined by TTC staining (P<0.05, Figure 5D). The AAR/LV ratio showed no significant statistical difference between groups, it indicates the good homogeneity of surgery. These data suggest that silencing of XIST regulates myocardial infarction and participates in mediating the signal for cell death in the heart.

**Figure 6:**
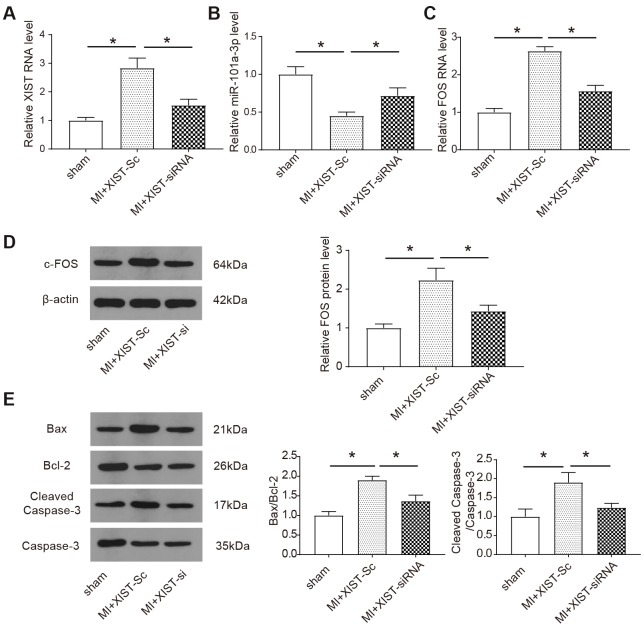
**Knockdown of lncRNA XIST regulates miR-101a-3p and FOS expressions and apoptosis-related proteins *in vivo.*** (**A**–**C**) The expression of XIST, miR-101a-3p and *FOS* was measured by qRT-PCR. The expression of myocardial infarction mice treated with XIST-siRNA adenoviruses was reduced, knockdown of XIST increases the expression of miR-101a-3p and reduces the expression levels of FOS in the infarct zone. (**D**) The protein expression level of c-FOS was detected by western blot. Knockdown of XIST reduces the expression of c-FOS (n=6). (**E**) The protein expression level of the apoptosis markers include Bax, Bcl-2, caspase 3 and cleaved caspase 3 was detected by western blot (n=6). Data are shown as mean ± SD, * *P<0.05*.

Subsequently, the expression of XIST, FOS and miR-101a-3p also the apoptosis-related proteins after inhibition of XIST were further detected *in vivo*. The expression of XIST, miR-101a-3p and *FOS* in the infarct area of the mice heart was measured by qRT-PCR. Compared to sham-operated animals, XIST and *FOS* were significantly upregulated in infarct heart tissues, while the XIST siRNA transfection suppressed the upregulation of XIST and *FOS* (*P*<0.05, Figure 6A, 6C). The same result was also verified in c-FOS protein expression level by western blot (Figure 6D). In contrast, the expression level of miR-101a-3p was significantly decreased as a consequence of MI and XIST siRNA transfection improved the level miR-101a-3p in infract heart tissues (*P*<0.05, Figure 6B). The protein level of apoptosis-associated proteins were measured by western blot. The expression of pro-apoptotic protein Bax and cleaved caspase-3 [24] was increased in infract heart tissues, while *in vivo* XIST siRNA transfection decreased the upregulation of these proteins (*P*<0.05, Figure 6E). Based on this part of the experimental results, we concluded that decreasing XIST expression could protect mice from myocardial infarction and cardiomyocyte apoptosis in some degree by down-regulating the expression of c-FOS and apoptosis-related proteins.

## DISCUSSION

The present study established that the lncRNA XIST was a damaging factor in MI, whereas miR-101a-3p was a protective miRNA in MI. The reciprocal alterations in the expression of these two non-coding RNAs (upregulation of XIST and downregulation of miR-101a-3p) in the infarct zones of MI hearts were observed. The opposite biological function and the expression level of XIST and miR-101a-3p could be well explained by ceRNA regulation, in which the XIST acts as a sponge to limit the level of miR-101a-3p. We proved that knocking down of XIST significantly improved the cell viability and decreased apoptosis rate under anoxia, and our results showed that the mice treated with XIST-siRNA adenoviruses exhibited a significant reduction in myocardial infarction sizes and the expression of apoptosis-related proteins. To put it in a nutshell, XIST regulates myocardial infarction and myocardial cell apoptosis through targeting the miR-101a-3p/FOS pathway.

Cardiovascular disease, especially MI, is one of the leading causes of death worldwide and its morbidity and mortality are on the rise year by year [25]. The epidemiological studies of MI demonstrated that age, male gender, smoking, elevated blood pressure, diabetes, obesity and a sedentary lifestyle each led to higher risk of MI occurrence [5]. Although there are more research results on myocardial infarction, there is still a lack of effective treatment for myocardial infarction. Thus, it is necessary to explore the new therapeutic approaches at the molecular level. Sufficient studies have shown that lncRNAs participates in a variety of human diseases by acting as a sponge, which reduces the expression level of miRNAs and correspondingly increases the expression level of target genes [26]. In the past decade, LncRNAs has attracted worldwide attention for its active involvement in various physiological and pathological processes of the heart [27]. The lncRNAs and miRNAs have been proven to be closely related to myocardial infarction [28, 29], myocardial autophagy [30, 31], atherosclerosis [32, 33] and other coronary artery disease pathogenesis [34, 35]. For example, Wang et al. revealed that the a long noncoding RNA, named autophagy promoting factor (APF) promotes autophagy and myocardial infarction by targeting miR-188-3p, they proved that the inhibition of miR-188-3p expression increased the expression of ATG7, which leading to increased myocardial infarction sizes in response to ischemia/reperfusion injury *in vivo* [36]. In another study, the downregulation of lncRNA 2810403D21Rik/Mirf (myocardial infarction-regulatory factor) was found to alleviate cardiac injury via up-regulating miR-26a, which targeted the Usp15 (ubiquitin specific peptidase 15) [37]. Many reports have indicated that lncRNA XIST plays critical role in cell proliferation, differentiation, and genome maintenance [15, 16], and we noticed that LncRNA XIST has been reported to be associated with myocardial infarction and cardiac hypertrophy [38–40]. However, there is no further information about the role of XIST in cardiovascular disease or other molecular mechanisms. In the present study, we revealed the lncRNA-miRNA-mRNA axis that participate in myocardial infarction, which was the XIST-miR-101a-3p-FOS axis.

Abnormal phenomena of miRNAs regulation appear in various diseases [41], and he aberrantly expressed miRNAs often participate in the pathogenesis of a certain disease [42, 43]. The expression of miR-101 has been reported to be reduced in the cardiac tissue of heart diseases, including aortic stenosis [44], dilated cardiac myopathy [44] and ischemic heart disease [42]. It has been frequently reported that the lncRNA XIST interacts with miRNAs to regulate cell function in different diseases, including cardiac diseases [45–47]. Chen et al. have proved that long non-coding RNA XIST promotes gastric cancer progression by acting as a molecular sponge of miR-101 to modulate EZH2 expression [47]. On the other hand, the study conducted by Xiao et al. revealed that XIST promoted cardiomyocyte hypertrophy by targeting miR-101, which enhanced the expression of toll-like receptor 2 (TLR2) [39]. In consistent with these findings, here we reported that suppression of XIST improved the viability of NMCMs that deprived of oxygen and reduced myocardial infarction area due to MI in the hearts of mice. Above all, we also identified that lncRNA XIST directly binding to miR-101a-3p to inhibit its activity. FOS and 3 other members, FOSB, FOSL1 and FOSL2, belong to the *Fos* gene family, which encode leucine zipper proteins that dimerize with JUN family proteins. The dimers can form a transcription factor complex with activator protein-1 (AP-1) [48–50]. Proteins encoded by *Fos* genes play an important role in cell differentiation, transformation, proliferation and apoptosis [51–53]. Ola et al. demonstrated that suppressing the activity of AP-1 in cardiac hypertrophy attenuated the secretion of agonist-induced atrial natriuretic peptide [54]. In addition the DNA-binding activity of AP-1 significantly increased in the ischemic myocardium (IM), septal wall (SW) and right ventricular wall after coronary ligation [55, 56]. Yang et al. has reported the overexpression of miR-101a can mitigate interstitial fibrosis and the deterioration of cardiac performance in postinfarct rats by targeting *FOS* [22]. The targeting relationship between miR-101a-3p and *FOS* has been confirmed in our experimental results, while our findings have also been confirmed in other reports [57, 58].

In conclusion, our present study reveals that lncRNA XIST is integrated into the machinery of myocardial infarction, and that XIST can affect myocardial infarction and cardiomyocyte apoptosis by regulating the expression of FOS. Moreover, we demonstrated that XIST acts as endogenous sponge RNA and suppresses miR-101a-3p expression and its molecular biological function via ceRNA regulation. Thus, modulation of XIST may represent a novel approach for interventional treatment of heart disease. This finding may provide a new clue for the understanding of lncRNA-controlled cellular events. We will further explore and study the potential regulation of lncRNA-miRNA interaction in future work, which may provide new candidates for effective treatment of heart disease.

## MATERIALS AND METHODS

### Cell culture and treatment

Experimental protocols for C57BL/6 mice were approved by the Animal Research Committee of the Zhengzhou University. Neonatal mice (C57BL/6, 1 to 3-day old) were acquired from the First Affiliated Hospital of Zhengzhou University. Mice were anesthetized by 4-5% isoflurane inducted through inhalation apparatus. Hearts were isolated and finely minced in HEPES-buffered saline solution. Thereafter, heart tissues were placed in incubations at 37°C, which contained HEPES-buffered saline solution with 0.14 mg/ml collagenase and 1.3 mg/ml pancreatin. After incubation, supernatants were collected, which were then centrifuged for 5 minutes at 200 g. After centrifugation, cells were placed in Dulbecco’s modified Eagle medium/F-12 (Gibco) for re-suspension, which contained 0.1 mM ascorbate, insulin-transferring sodium selenite media supplement (Sigma, St. Louis, MO, USA), 5% heat-inactivated horse serum, 100 U/ml penicillin and 100 μg/ml streptomycin. The dissociated cells were pre-plated for 1 hour at 37 °C. After 1 hour, cells were diluted to 1 × 10^6^ cells/ml and plated in 10 μg/ml laminin-coated culture dishes. Dispersed NMCMs were cultured in Dulbecco’s modified Eagle’s medium (DMEM) containing 10% fetal bovine serum in a 5% CO2 and 37°C humidified atmosphere. To induce hypoxia condition, NMCMs were incubated in a hypoxic chamber for 2-8 hours (5% CO_2_ and 95% N_2_).

### qPT-PCR

Total RNA in cells or tissues was extracted and separated with TRIzon reagent (Cwbiotech, CW0580) from lysates. For each sample, 500 ng of total RNA was converted to cDNA using High Capacity cDNA Reverse Transcription Kit (Transgene, AT341-02). The relative expression levels of mRNAs, miRNAs and lncRNAs were quantified by qRT-PCR with SYBR Green I (Roche, 4913914001). Detection of the qRT-PCR results was performed on CFX96 Real-Time PCR Detection System (Bio-Rad). All of the primers are provided in Supplementary Table 1. The level of miR-101a-3p was analyzed by qRT-PCR normalizing with U6, while the levels of lncRNA and mRNA |were normalized with glyceraldehyde-3-phosphate dehydrogenase (GAPDH).

### Cell transfection

NMCMs were seeded in a 96-well culture plate at a density of 1x105 cells/well (200 μl/well and 6 wells for each group). Cells were washed twice with phosphate-buffered saline (PBS) before being transfected. To knock down XIST, cells were transfected with 50 nM XIST siRNAs (S1, S2 or S3) or scramble form (Sc) as control. Cells were kept in the culture for 48 hours. To study the role of miR-101a-3p, NMCMs were transfected with 40 nM miR-101a-3p mimic or miR-101a-3p inhibitor for 48 hours. The transfection efficiency was exanimated by qRT-PCR. The siRNAs and miR-101a-3p mimic or inhibitor were transfected into cells using Lipofectamine 2000 (Invitrogen, 11668030) according to the manufacturer’s instructions. All siRNA, mimics and inhibitors sequences are listed in Supplementary Table 2.

### Proliferation detection with MTT assay

Myocardial cells were seeded in a 96-well culture plate (200 μl/well and 6 wells for each group) at a density of 1x10^5^ cells/well. After 48 h of transfection and 8 h anoxia treatment, the cells were incubated with 20 μl MTT (Sigma, M2128) (0.5 mg/ml) and 180 μl DMEM for 4 h. Then, the MTT solution was removed and 150 μl of dimethyl sulfoxide (DMSO) was added to each well. The absorbance of each well was examined at 490 nm with a microplate reader.

### Determination of apoptosis levels

The apoptosis levels were determined with flow cytometry (BD, San Jose, USA), using an Annexin V-fluorescein isothiocyanate (FITC) apoptosis detection kit (Beyotime Institute of Biotechnology, C1062L) in the light of the manufacturer's instructions. The cells in the treated groups were transfected with 50 nM XIST siRNAs or 40 nM miR-101a-3p mimic, and the untreated groups served as the siRNA control or mimic control. After 48 h under anoxia conditions, the cells were harvested and washed twice with phosphate buffered saline (PBS). Then the cells were dyed with 1 ml of Annexin V binding buffer containing 10 μl of a propidium iodide (PI) solution and 5 μl of AnnexinV-FITC for 10 min at room temperature in the dark. All experiments were performed in triplicate.

### Bioinformatics analysis

Analysis of target relationships between XIST and miR-101a-3p was performed using StarBase v2.0, and the relationship between *FOS* and miR-101a-3p was assessed using http://www.microrna.org/.

### Luciferase reporter assay

XIST 3’-UTR was amplified by PCR. The primers provided in Supplementary Table 1. To produce mutated 3’-UTR, the QuikChange II XL Site-Directed Mutagenesis Kit (Stratagene, 200522) was used to generate the mutations. Wild type and mutated 30UTRs were subcloned into the pGL3 vector (Youbio, VT1556) immediately downstream the coding region of luciferase gene. Mouse XIST wild type (APF-wt), FOS wild type (FOS-wt) and the mutant derivative devoid of miR-101a-3p binding site (APF-mut, FOS-mut) were cloned downstream the coding region of luciferase gene. NMCMs were infected with the indicated adenoviruses, then transfected with the indicated luciferase constructs. The Lipofectamine 2000 (Invitrogen, 11668030) was used for transfection according to the manufacturer’s instruction. Luciferase activities were assessed using a Luciferase Reporter Assay System (Promega), and each transfected well was detected in triplicate.

### Pull down assay

NMCMs were transfected with 50 mM biotinylated miRNA for 48 h. The cells were washed with PBS followed by brief vortex and incubated in a lysis buffer (Invitrogen, EPX-99999-000). The lysates were precleared by centrifugation and 50 ml of the samples were aliquoted for input. The remaining lysates were incubated with streptavidin agarose beads (Invitrogen, 35137). The beads were incubated at 4 °C for 3 h, were washed briefly three times and boiled in sodium dodecyl sulfate (SDS) buffer. The RNA present in the pull-down material was detected using reverse-transcription polymerase chain reaction (RT-PCR) and quantitative real-time PCR (qPCR) analysis. The primer pairs were provided in Supplementary Table 1.

### RNA immunoprecipitation (RIP) assay

The RIP assay was performed in the RNA Immunoprecipitation (RIP) Kit (sigma, 17-701) according to manufacturer’s instruction. At room temperature, magnetic beads were firstly incubated with anti-AGO2 antibodies (cell signaling technology, #2897) for 30 min. Then, the cells were lysed in RIP lysis buffer and cell lysates were immunoprecipitated with magnetic beads at 4 °C for 6 h. Thereafter, RNA was purified and measured by qRT-PCR.

### Western blot

Total protein was extracted from the samples using 100 μl radio immunoprecipitation assay lysis buffer (RIPA). Lysates were centrifuged at 4°C for 20 min and the supernatants were collected. The protein concentration was determined by the BCA (Bicinchoninic acid) assay. Protein samples (20 μg) were loaded and electrophoresed using dodecyl sulfate, sodium salt (SDS)-polyacrylamide gel electrophoresis at 90 V for 1.5 h and subsequently transferred to polyvinylidene fluoride (PVDF) membranes. Membranes were sealed for 1 h in 5% BSA. Slides were incubated with primary antibodies diluted in Tris Buffered Saline and Triton (TBST) at 4°C overnight. The primary antibodies used were anti-c-*FOS* (1:1000, Abcam, ab190289), anti-Bax (1:1000, Abcam, ab32503), anti-Bcl-2 (1:1000, Abcam, ab182858), anti-caspase 3 (1:1000, Abcam, ab13847), anti-cleaved caspase 3 (1:1000, Abcam, ab49822) and anti-β-actin (1:5000, Abcam, ab8227). Membranes were washed with TBST 3 times, and then, horseradish peroxidase-conjugated (HRP) secondary antibodies were added for each corresponding primary antibody species. β-actin served as the loading control.

### Adenoviral constructions and infection

Male 8-week-old C57BL/6 mice were acquired from the First Affiliated Hospital of Zhengzhou University and were randomly allocated into sham-operated group (sham) and myocardial infarction groups. The use of animals was authorized and informed by the First Affiliated Hospital of Zhengzhou University. Specific small mice interfering RNA (siRNA) XIST of mice and scrambled oligonucleotides were designed and synthesized by GenePharma (Shanghai, China). XIST siRNA or control sequence was inserted into an adenovirus vector by using the pSilencer adeno 1.0-CMV System (Ambion) in accordance to the manufacturer’s instructions. According to methods previously described [11]. Adenoviral infection of cardiomyocytes was performed as previously described [59]. We performed the intracoronary delivery of adenoviruses. Mice were anesthetized and the chest was entered through a small left anterior thoracotomy. The main pulmonary artery and ascending aortic artery were clamped. Then, 2×10^11^ moi adenovirus carrying XIST siRNA (Adv-si) or a scrambled control (Adv-Sc) was injected with a catheter from the apex of the left ventricular into the aortic root with a syringe. After that, mice were placed into cage for recovery. Mice in the sham group underwent the same procedures.

### Myocardial infarction model

Five days after adenoviruses transfection, MI models were established as described [60]. After anesthetization with 40 mg/kg sodium pentobarbitone (i.p.) and 12.5 mg/kg xylazine (i.p.), the upper limbs of mice were taped to the table, placed in the supine position. Along the left side of the mice sternum, an incision of approximately 1-1.5 cm in length was made. To avoid bleeding, the muscle layers of the chest wall were bluntly dissected. At the point of the most pronounced cardiac pulsation, the thorax was opened, and the right side of the chest was pressed to push the heart out of the thoracic cavity. After occluding the left anterior descending coronary artery, the chest was closed. The whole surgical procedure was performed under sterile conditions. Mice in the sham group underwent open-chest procedures without coronary artery occlusion. After 24 h, mice were anesthetized with sodium pentobarbitone (40 mg/kg, i.p.) and xylazine (12.5 mg/kg, i.p.) and sacrificed with their hearts quickly isolated. The procedures were in accordance with the regulations of the Ethics Committee of First Affiliated Hospital of Zhengzhou University and the Guide for the Care and conformed to the Guide for the Care and Use of Laboratory Animals published by the US National Institutes of Health (NIH Publication No. 85–23, 2011).

### TTC staining

After successfully constructing the MI mice model, the left ventricle was injected with 1% Evans blue (1 ml) (Evans Blue, E2129-10G), then mice were sacrificed and the heart was rapidly excised. The heart slices were incubated in 1% 2,3,5-triphenyltetrazolium chloride (TTC) (Sigma, T8877) for 15 min at 37 °C for demarcation of the viable and nonviable myocardium within the risk zone. To determine the homogeneity of surgery and the severity of ischemia injury, the AAR/LV ratio (area at risk/left ventricle weight) and INF/AAR ratio (infarct size/area at risk) were calculated respectively [11, 61].

### Statistical analysis

Data were expressed as mean ± SD of at least three independent experiments for each cellular experimental group and at least six independent experiments for each animal group. All experimental data were analyzed using SPSS (version 20.0) and using GraphPad Prism 7.0 unless otherwise stated. Then, we evaluated the data with Student’s t-test for comparison between two groups and one-way analysis of variance (one-way ANOVA) for comparison among multiple groups (>2). A value of P <0.05 was considered to be statistically significant.

### Ethical approval

All procedures performed in studies involving animals were in accordance with the ethical standards of the First Affiliated Hospital of Zhengzhou University. This article does not contain any studies with human participants performed by any of the authors.

## Supplementary Material

Supplementary Tables
