# Supplementary material for: LncRNA XIST promotes myocardial infarction by regulating FOS through targeting miR-101a-3p
Source: Aging (Albany NY). 2020 Apr 21;12(8):7232–47. doi: 10.18632/aging.103072 (PMC7202499; doi:10.18632/aging.103072)
Supplement: Supplementary Tables [file aging-12-103072-s001..pdf]

SUPPLEMENTARY TABLES

Supplementary Table 1. Primer sequences for PCR and qRT-PCR.

Gene	Forward primers (5'→3')	Reverse primers (5'→3')
Mmu-XIST	GTCAGCAAGAGCCTTGAATTG	TTTGCTGAGTCTTGAGGAGAATC
Mmu *c-Fos*	TGGCTGGTGCAGCCCACTCT	CTTCACCATTCCCGCTCTGGCG
Mmu-GAPDH	GGG AAGCCCATCACCATCTTC	AGAGGGGCCATCCACAGTCT
Mmu-miR-101a-3p	TGGGCTA CAGTACTGTGATA	TGCGTGTCGTGGAGTC
Mmu-U6	CGCTTCACGAATTTGCGTGTCAT	GCTTCGGCACATATACTAAAAT

Supplementary Table 2. Regent for transfection.

Regent	Sense (5'→3')	Antisense (5'→3')
XIST siRNA1	GCACUUCUCUUGUCAAUAU	AUAUUGACAAGAGAAGUGC
XIST siRNA2	GCUCUUCAUUUGGACUUAA	UUAAGUCCAAAUGAAGAGC
XIST siRNA3	AUAACAGUAAGUCUGAUAGAGGACA	UGUCCUCUAUCAGACUUACUGUUAU
siRNA control	UUACUCAUGUGUCAUAACACAGGUG	CACCUGUGUUAUGACACAUGAGUAA
miR-101a-3p mimics	UACAGUACUGUGAUAACUGAA	UUCAGUUAUCACAGUACUGUA
NC mimics	UUCUCCGAACGUGUCACGUUT	ACGUGACACGUUCGGAGAATT
miR-101a-3p inhibitor	TTATCACAGTACTGT	
NC inhibitor	ACGTCTATACGCCCA	
